# Efficacy and safety of yoga for the management of chronic low back pain: an overview of systematic reviews

**DOI:** 10.3389/fneur.2023.1273473

**Published:** 2023-10-27

**Authors:** Xianshuai Zhang, Tianying Chang, Wenlong Hu, Mingpeng Shi, Yating Chai, Siyi Wang, Guohui Zhou, Mingze Han, Minghui Zhuang, Jie Yu, He Yin, Liguo Zhu, Changwei Zhao, Zhenhua Li, Xing Liao

**Affiliations:** ^1^Changchun University of Chinese Medicine, Changchun, Jilin, China; ^2^Affiliated Hospital of Changchun University of Chinese Medicine, Changchun, Jilin, China; ^3^Shenzhen People's Hospital, Shenzhen, China; ^4^Department of Radiology, The First Hospital of Jilin University, Changchun, Jilin, China; ^5^China Academy of Chinese Medical Sciences, Wangjing Hospital, Beijing, China; ^6^Center for Evidence-Based Chinese Medicine, Institute of Basic Research in Clinical Medicine, China Academy of Chinese Medical Sciences, Beijing, China

**Keywords:** chronic low back pain, systematic review, GRADE, AMSTAR-2, yoga

## Abstract

**Background:**

Yoga is a non-pharmacological conservative therapeutic modality that can be employed for the management of chronic low back pain (CLBP). In this overview, we have summarized and evaluated data from current systematic reviews (SRs) on the use of yoga for CLBP.

**Methods:**

We comprehensively searched SRs on the use of yoga for CLBP in nine electronic databases from inception to September 2023. The methodological quality was evaluated using the Assessment of Multiple Systematic Review Scale-2 (AMSTAR-2). The reporting quality of the included SRs was evaluated using the Preferred Reporting Item for Systematic Review and Meta-Analysis-2020 (PRISMA-2020), and the quality of data was graded using the Grading of Recommendations Assessment, Development, and Evaluation (GRADE). Two independent researchers performed the screening, data extraction, and quality assessment process of SRs.

**Results:**

A total of 13 SRs were included. The results of the AMSTAR-2 indicated that the methodological quality of the included studies was relatively low. The PRISMA-2020 checklist evaluation results indicated that methodological limitations in reporting, especially regarding data processing and presentation, were the main weaknesses. The GRADE assessment indicated that 30 outcomes were rated moderate, 42 were rated low level, and 20 were rated very low level. Downgrading factors were mainly due to the limitations of the included studies.

**Conclusion:**

Yoga appears to be an effective and safe non-pharmacological therapeutic modality for the Management of CLBP. Currently, it may exhibit better efficacy in improving pain and functional disability associated with CLBP. However, the methodological quality and quality of evidence for SRs/MAs in the included studies were generally low, and these results should be interpreted cautiously.

## 1. Introduction

Low back pain (LBP) is a prevalent clinical concern and symptom, which is defined as pain or discomfort in the area between the lower rib and the gluteal folds (1). The global population prevalence rate of LBP has reached 7.3% (2). The lifetime prevalence rate of LBP can be as high as 47% in low-income countries such as Africa (3). A systematic review shows that running can decrease the incidence of LBP and can serve as a protective factor for preventing the onset of LBP (4). The etiology of LBP is complex and not completely understood; neurological, bladder dysfunction, loss of anal sphincter tone, and saddle anesthesia are factors that can contribute to the onset of LBP (5). Clinicians can identify potential pathologies that resemble musculoskeletal conditions through the use of screening and differential diagnosis. More emphasis should be placed on patients with signs and symptoms that resemble severe pathology in the thoracolumbar region, such as LBP due to post-traumatic thoracolumbar fracture (6), and other potential non-musculoskeletal causes of LBP including LBP due to secondary peripheral arterial disease (7). Chronic low back pain (CLBP) is LBP lasting 3 months or longer. More than 70% of people experience CLBP at least once in their lifetime (8). CLBP can cause physical diseases and lead to anxiety and depression, thus decreasing the quality of life (9). CLBP has become a significant public health concern. The resulting inability to work, disability, and medical expenditure have imposed a substantial financial burden on individuals and society (10).

Presently available biomedical therapies for CLBP are expensive, have poor long-term efficacy, and may cause adverse side effects (11). Therefore, many patients with CLBP prefer alternative treatments. Recent practice guidelines from the American College of Physicians suggest that non-pharmacological therapeutic modalities can be considered if standard medical treatments fail to alleviate LBP (12). Exercise is strongly recommended as a non-pharmacological intervention because it can effectively relieve pain (13). Yoga has garnered widespread attention as a characteristic meditative movement therapy that integrates body and mind (14). Yoga originated in ancient India and has a history of over 4,000 years. Yoga comprises several key components, such as physical posture (asana), controlled breathing techniques (pranayama), relaxation, and meditation (dhyana) (15). The inherent nature of yoga is characterized as gentle and soothing. It can improve the strength of the back muscles and alleviate pain while promoting the extension, flexibility, and balance of the body tissue of the spinal vertebra (16). Yoga can improve physical functions. One study has shown that yoga can decrease anxiety and improve self-efficacy and pain acceptance (17). This is particularly helpful because CLBP occurs due to a complex interplay of biological, psychological, and social factors, and the availability of emotional support from practicing yoga can improve the confidence of the patients in overcoming the disease (18). Furthermore, the underlying mechanism of action of yoga is linked to contextual factors, which are the result of a combination of personal, disease-related, and environmental factors (19), where pain-induced contextual factors can be conceptualized as triggers of placebo and nocebo effects (20). Particularly in musculoskeletal disorder (MSK)-associated pain, the mood and expectations of the patient can affect MSK pain (21). Previous studies have shown that physiotherapy for MSK can effectively ameliorate pain when patients redirect their attention away from the disease compared with when patients focus on the pain (22).

Many clinical studies, systematic reviews (SRs), and reviews have reported the efficacy of yoga in the treatment of CLBP. However, adequate and unified data are not available. An overview of SRs can be performed to comprehensively collect and evaluate the relevant systematic evaluation of the treatment, etiology, diagnosis, and prognosis of the same disease or health problem. This can provide more robust, high-quality evidence for clinicians and promote their decision-making ability (23). In this study, we evaluated and objectively summarized the efficacy and safety of yoga in treating CLBP by overviewing SRs to provide clinicians with evidence of synthesis that can serve as a basis for decision-making.

## 2. Methods

This overview was performed according to the Cochrane Handbook for SRs of Interventions (24) and the Preferred Reporting Items for Systematic Reviews and Meta-Analyses (PRISMA) (25).

### 2.1. Inclusion and exclusion criteria

Inclusion criteria for the overview were established using the Population, Intervention, Comparator, Outcome, and Study design (PICOS) framework, which were as follows: (a) Participants: Adults over 18 years of age who are diagnosed with CLBP and patients diagnosed with chronic non-specific LBP based on the LBP diagnostic criteria were included (1). CLBP refers to low back pain over 3 months, and 85% of chronic back pain was non-specific, with no clear pathoanatomic explanation. (b) Interventions: yoga or combined with other therapies. (c) Comparator: Treatments other than yoga, such as other exercise therapy, placebo, health education, and blank control, to fulfill the research conditions. (d) Outcomes: the primary outcome was pain relief. Secondary outcomes included disability function, quality of life, and adverse effects of yoga for managing CLBP. (e) Study design: SRs with or without meta-analysis (MAs) of randomized controlled trials (RCTs) were included. In these studies, yoga was used as a treatment modality for managing CLBP.

Exclusion criteria for the overview were as follows: (a) incomplete information or incorrect data in a systematic review, (b) duplicated SRs/MAs, (c) for updated reviews, non-latest works of literature will be excluded, (d) systematic review with network meta-analysis or indirect comparison, and (e) dissertation or conference papers.

### 2.2. Search strategy

Computer searches of PubMed, EMBASE, Cochrane Library, Web of Science, Physiotherapy Evidence Database (PEDro), China Knowledge Network (CNKI), VIP, Wanfang Database, and Chinese Biomedical Databases (CBM) were conducted to collect systematic evaluations of yoga for CLBP. The retrieval time has been updated from the database inception to September 2023. The search used a combination of subject headings and free words. Key phrases included “yoga,” “low back pain,” “back pain,” “lumbar disc herniation,” “meta-analysis,” or “systematic review.” Furthermore, we also searched conference abstracts and reference lists of all retrieved articles to avoid missing relevant SRs/MAs. The search strategy for PubMed is shown in Table 1. More search strategies are mentioned in Appendix 1.

**Table 1:** Search strategy for PubMed.

**Query**	**Search term**
#1	Yoga [MeSH Terms]
#2	(yogic [Title/Abstract])) OR (yogi [Title/Abstract])) OR (yog^*^[Title/Abstract]))
#3	#1 OR #2
#4	Low Back Pain [Mesh]
#5	Low back pain[Title/Abstract]) OR (low back pains[Title/Abstract])) OR (lumbago[Title/Abstract])) OR (lower back pain[Title/Abstract])) OR (lower back pains[Title/Abstract])) OR (low back ache[Title/Abstract])) OR (low back aches[Title/Abstract])) OR (low backache[Title/Abstract])) OR (low backaches[Title/Abstract])) OR (lumbar pain[Title/Abstract])) OR (herniated disk[Title/Abstract])) OR (herniated disc[Title/Abstract])) OR (hernia intervertebral disc[Title/Abstract])) OR (lumbar degenerat^*^[Title/Abstract])) OR (backache[Title/Abstract])) OR (back disorders[Title/Abstract])) OR (sciatica[Title/Abstract])) OR (coccyx[Title/Abstract])) OR (coccy^*^[Title/Abstract])) OR (spondylosis[Title/Abstract])
#6	#4 OR #5
#7	“Systematic Review” [Publication Type]) OR (“Systematic Reviews as Topic”[Mesh])) OR (“Meta-Analysis” [Publication Type])) OR (“Meta-Analysis as Topic”[Mesh])) OR (Systematic review [Title/Abstract])) OR (Meta-analysis [Title/Abstract])
#8	#3 AND #6AND #7

### 2.3. Study selection and data abstraction

The systematic review literature obtained from the search was imported into NoteExpress. Two reviewers (XS-Z and TY-C) independently performed two rounds of screening by reading the title, abstract, and complete text. Any disagreements among the reviewers were resolved by discussion or by consulting with an experienced, authoritative third reviewer (XL) to reach a final decision. The content of data extraction included author, year, publication language, number of included studies, sample size, intervention and control measures, quality assessment tool, outcome indicators, and principal conclusions.

We retrieved the original research studies for each system evaluation using an Excel spreadsheet and used the Guidance for the Review of Overviews of Reviews (GROOVE) to evaluate the degree of overlap. OVErviews (GROOVE) (26) is a user-friendly tool, wherein the matrices of evidence and the calculation of the corrected covered area (CCA) are one of the most exhaustive methods for measuring overlap. The CCA value from 0 to 5 represents slight overlap, 6–10 represents moderate overlap, 11–15 represents high overlap, and >15 represents a very high degree of overlap.

### 2.4. Quality appraisal and assessment of evidence

Two trained and qualified reviewers, XS-Z and TY-C, used the Assessing the Methodological Quality of Systematic Reviews 2 (AMSTAR-2), Preferred Reporting Item for Systematic Review and Meta-Analysis-2020 (PRISMA-2020), and Grading of Recommendations Assessment, Development, and Evaluation (GRADE) to evaluate the methodological, reporting, and evidence quality of the included studies, respectively. Any disagreements between the two reviewers were resolved by consulting an experienced, authoritative third reviewer (XL).

#### 2.4.1. Methodological assessment tool—AMSTAR-2 scale

AMSTAR-2 (27) was used to evaluate the quality of the methodology included in SR, which contains 16 items. Each item was rated as “yes,” “partially yes,” and “no,” and the methodological quality was divided into four categories of “high,” “moderate,” “low,” and “very low” based on the evaluation results of the grade of the key items (items 2, 4, 7, 9, 11, 13, and 15). AMSTAR-2 scale categorized the methodological quality of the systematic evaluation/meta-analysis into the following four levels: (a) high quality, characterized as no or one non-critical weakness; (b) moderate quality, characterized as more than one non-critical weakness; (c) low quality: characterized as one critical flaw with or without non-critical weaknesses; and (d) critically low quality, characterized as more than one critical flaw with or without non-critical weaknesses.

#### 2.4.2. Report quality assessment tool—PRISMA-2020 statement

PRISMA-2020 (28) was used to evaluate the reporting specifications in SRs, containing 27 main items and 42 sub-items. The complete report of each item was recorded as “1 point.” Some reports were recorded as “0.5 point,” and no report was recorded as “0 point.” A report with completeness of more than 80% (33–42 points) was considered “relatively complete” and rated as high quality. If the completeness of the report was above 60% (25–32 points), it was considered “the report has certain defects” and rated as medium quality. If the completeness of the report was below 60% (<25 points), it was considered a “relatively serious lack of information” and rated as low quality (29).

#### 2.4.3. Evidence quality assessment—GRADE system

The GRADE (30) was used to comprehensively evaluate the quality of the outcome indicators. Initially, RCT-derived evidence was considered to be of high quality; however, confidence in such evidence may decrease due to the following five factors: risk of bias, inconsistency, indirectness, imprecision, and publication bias. The quality of evidence was graded based on the confidence in approaching the estimated efficacy with real efficacy as follows: (a) high quality (very confident in efficacy); (b) medium quality (confidence was average, and a significant difference may be present between actual and estimated efficacy); (c) low quality (limited confidence, and a significant difference between actual and estimated efficacy); and (d) extremely low quality (with almost no confidence, and a significant difference may be present between actual and estimated efficacy).

## 3. Results

### 3.1. Literature search

**Figure 1:**
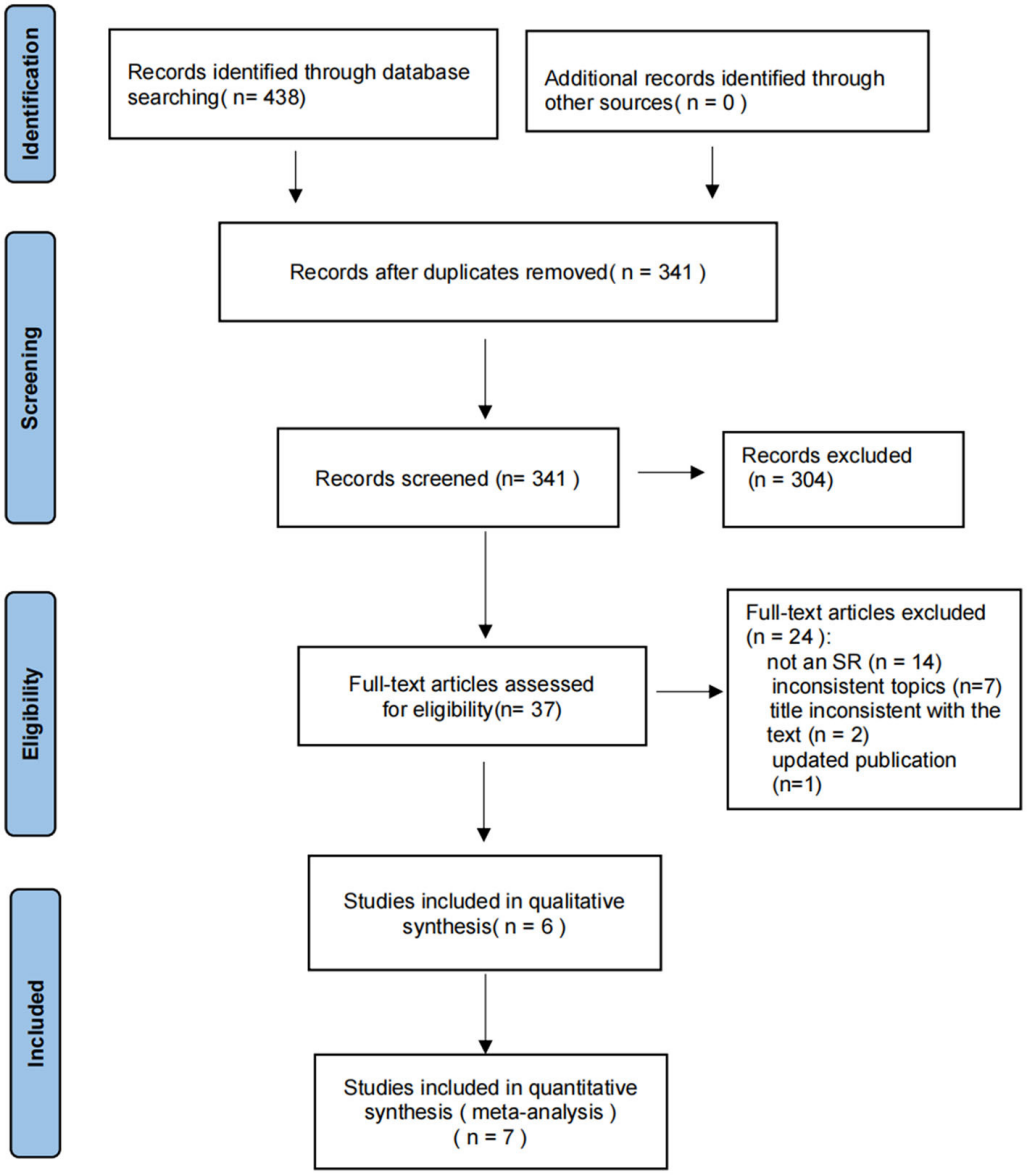
Flow diagram of the literature selection.

Based on the established search strategy, we performed a preliminary search and searched 438 articles across nine databases from database inception to September 2023. After eliminating duplicate 97 articles, we obtained 341 studies. We then screened the titles and abstracts and excluded 304 studies. The complete texts of the remaining 37 studies were read, and after a detailed review, 24 studies with no SR, inconsistent topics, titles inconsistent with the text, or updated publication were excluded. Finally, 13 SRs (31–43) fulfilled the inclusion criteria and were selected for analysis, which included seven MAs and six qualitative analyses. The process and results of the literature screening are shown in Figure 1.

### 3.2. Characteristics of SRs

The characteristics of the included SRs are shown in Table 2. Of the 13 SRs, two SRs (31, 32) were published in Chinese, whereas 11 SRs (33–43) were published in English. The publication date ranged from 2011 for the earliest SR to 2022 for the most recent. The number of RCTs included in each SR ranged from 4 to 27 and the number of patients participating in the RCTs ranged from 403 to 2,702. The methodological quality of the RCTs was assessed in all 13 SRs using different tools. Two SRs (31, 43) used the Jadad scale, one SR (40) used the PEDro scale, seven SRs (32–36, 39, 42) used the Cochrane risk of bias tool, and three SRs (37, 38, 41) used other methods. The interventions in the treatment groups primarily involved yoga, either alone or as a combination with other therapies. The control groups received physical exercise, education, usual care, or no treatment. A total of 12 SRs used pain as the endpoint outcome. A total of 11 SRs (31–34, 36–38, 40–43) used physical function and disability as the endpoint outcome, six SRs (31, 33, 34, 36, 40, 42) used quality of life as the endpoint outcome, and six SRs (33, 34, 36, 37, 42, 43) used adverse events as the endpoint outcome. All studies concluded that yoga can improve CLBP and functional disability to varying degrees.

**Table 2:** Characteristics of included systematic reviews.

**References**	**Country**	**No. of included RCTs**	**Interventions**	**Comparisons**	**Quality assessment tools**	**Outcomes**	**Overall conclusions**
Zhang and Hu (31)	China	19 (1,660)	Yoga	No treatment, placebo, other conservative therapy, or another exercise	Jada scale	Pain intensity, functional disability, quality of life	Yoga therapy can effectively alleviate patients' back pain and improve their dysfunction, and the curative effect is long-term
Kang et al. (32)	China	14 (1,684)	Yoga	Conservative therapy	Cochrane risk of bias tool	Functional disability	Available evidence shows that yoga can achieve better results in the treatment of lower back pain
Dennis et al. (33)	Germany	27 (2,702)	Yoga	No treatment, usual care, other passive treatments, or any active treatment	Cochrane risk of bias tool	Pain, back-specific disability, quality of life, adverse events	Yoga revealed robust short- and long-term effects for pain, disability, and physical function when compared to non-exercise controls and no significantly different effects when compared to exercise controls
Zhu et al. (34)	China	18 (1,852)	Yoga or in combination with other treatments	No treatment, a minimal intervention (e.g., education and booklets), usual care, or other active treatments	Cochrane risk of bias tool	Pain, disability, quality of life, adverse events	Yoga might decrease pain from the short term to the intermediate term and improve functional disability status from the short term to the long term compared with non-exercise (e.g., usual care and education)
Sang (35)	Korea	6 (523)	Yoga	No intervention, or any other intervention	Cochrane risk of bias tool	Pain intensity	Yoga programs could significantly reduce CNSLBP
Wieland et al. (36)	USA	21 (2,223)	Yoga	Any other intervention or no intervention	Cochrane risk of bias tool	Back-specific functional status, pain, clinical improvement, mental or physical quality of life, depression, adverse events	There is low- to moderate-certainty evidence that yoga compared to no exercise results in small and clinically unimportant improvements in back-related function and pain
Douglas et al. (37)	USA	10 (1,053)	Yoga	No treatment, another exercise, education, usual care	Evidence criteria	Physical function and disability, pain, psychological, adverse events	Yoga appears to be an effective and safe intervention for chronic low back pain
Susan and Beggs (38)	UK	8 (743)	Yoga	No treatment, another exercise, education, usual care	CLEAR NPT	Pain, functional disability	Yoga may be an efficacious adjunctive treatment for CLBP
Manoj and Haider (39)	USA	13 (1,386)	Yoga	No treatment, physical exercise, education, usual care, etc.	Cochrane risk of bias tool	Pain	Yoga as part of the intervention can be a reduction in low back pain
Alison et al. (40)	USA	10 (1, 024)	Yoga	No treatment, usual care, a self-care book, stretching, or other forms of exercise	PEDro scale	Pain, disability, quality of life	Evidence demonstrates moderate support for yoga as an effective treatment for LBP
Christopher (41)	UK	4 (711)	Yoga	Other care modalities	CASP reviewer checklist	Pain, functional disability	Yoga is an effective management tool for CLBP, it is effective in improving back function
Holger et al. (42)	Germany	10 (967)	Yoga	No treatment, usual care, or any active treatment	Cochrane risk of bias tool	Pain, back-specific disability, generic disability, health-related quality of life, adverse events	Yoga can be recommended as an additional therapy for chronic low back pain patients
Paul and Ernst (43)	UK	7 (403)	Yoga	Usual care, physical exercises, education, or no treatment	Jada scale	Pain, functional disability, Beck Depression Inventory, adverse events	Yoga has the potential to alleviate low back pain

LBP, low back pain; CLBP, chronic low back pain; CNSLBP, chronic non-specific lower back pain; CLEAR NPT, checkist to evaluate a report of a non-pharmacological trial; PEDro, Physiotherapy Evidence Database; CASP, Critical Appraisal Skills Programme.

### 3.3. Methodological quality of included SRs

The overall quality of the SRs was graded using AMSTAR-2 based on seven critical domains. Only one SR (36) was rated as high-quality evidence, two SRs (33, 34) were as low quality, and the remaining 10 SRs (31, 32, 35, 37–43) were rated as critically low quality. These SRs had deficiencies in the following key areas: three SRs (33, 34, 36) had registered protocols (Item 2), two SRs (36, 43) used comprehensive literature search strategies, the other 11 (31–35, 37–42) SRs had not searched gray literature (Item 4), only one SR (36) provided a list of excluded studies with reasons (Item 7), five SRs (31, 37, 39–41) did not use appropriate tools to assess the risk of bias (Item 9), six SRs (31, 37, 39–41, 43) did not perform meta-analysis (Item 11), five SRs (33, 34, 36, 40, 42) considered risk of bias in interpretations (Item 13), and finally six SRs (32–36, 42) assessed publication bias using funnel plots (Item 15). The absence of these key domains decreased the literature quality. For other items, 10 SRs (31–35, 38–40, 42, 43) did not report study settings and follow-up, which was rated as “Partial yes” for Item 8. Six SRs (31, 37, 39–41, 43) without meta-analysis were rated as “no meta-analysis” for Item 12. The main methodological limitations were the absence of a clear rationale for the inclusion criteria and a list of excluded studies. Details of evaluations for other items are shown in Table 3.

**Table 3:** Result of the AMSTAR-2 assessments.

**First author**	**Type of study/publication year**	**AMSTAR-2 quality items**																**AMSTAR-2 classification**
		**Q1**	Q2^*^	**Q3**	Q4^*^	**Q5**	**Q6**	Q7^*^	**Q8**	Q9^*^	**Q10**	Q11^*^	**Q12**	Q13^*^	**Q14**	Q15^*^	**Q16**	
Zhang	SR/2016	Y	N	N	PY	Y	N	N	PY	PY	N	NM	NM	N	N	NM	N	Critically low
Kang	SR/MA/2020	Y	N	N	PY	Y	Y	N	PY	Y	N	Y	N	N	Y	Y	N	Critically low
Dennis	SR/MA/2022	Y	Y	N	PY	N	Y	N	PY	Y	Y	Y	Y	Y	Y	Y	Y	Low
Zhu	SR/MA/2020	Y	Y	N	PY	Y	Y	N	PY	Y	N	Y	Y	Y	Y	Y	N	Low
Sang	SR/MA/2020	Y	N	N	PY	N	N	N	PY	Y	N	N	N	N	N	Y	N	Critically low
Wieland	SR/MA/2022	Y	Y	N	Y	Y	Y	Y	Y	Y	Y	Y	Y	Y	Y	Y	Y	High
Douglas	SR/2016	Y	N	N	PY	N	N	N	N	N	N	NM	NM	N	N	NM	N	Critically low
Susan	SR/MA/2013	Y	N	N	PY	N	N	N	PY	Y	N	Y	Y	N	Y	N	N	Critically low
Manoj	SR/2013	N	N	N	PY	N	N	N	PY	N	Y	NM	NM	N	N	NM	Y	Critically low
Alison	SR/2013	N	N	N	PY	Y	N	N	PY	Y	N	NM	NM	Y	N	N	N	Critically low
Christopher	SR/2013	Y	N	N	N	N	N	N	N	N	N	NM	NM	N	N	NM	N	Critically low
Holger	SR/MA/2013	Y	N	N	PY	N	Y	N	PY	Y	N	Y	N	Y	N	Y	N	Critically low
Paul	SR/2011	N	N	N	Y	N	N	N	PY	Y	N	NM	NM	N	Y	NM	N	Critically low

SR, systematic review; Y, yes; N, no; PY, partial yes; MA, meta-analysis; NM, no meta-analysis.

Q1: Did the research questions and inclusion criteria for the review include the components of PICO?

Q2^*^: Did the report of the review contain an explicit statement that the review methods were established before the conduct of the review and did the report justify any significant deviations from the protocol?

Q3: Did the review authors explain their selection of the study designs for inclusion in the review?

Q4^*^: Did the review authors use a comprehensive literature search strategy?

Q5: Did the review authors perform study selection in duplicate?

Q6: Did the review authors perform data extraction in duplicate?

Q7^*^: Did the review authors provide a list of excluded studies and justify the exclusions?

Q8: Did the review authors describe the included studies in adequate detail?

Q9^*^: Did the review authors use a satisfactory technique for assessing the risk of bias (RoB) in individual studies that were included in the review?

Q10: Did the review authors report on the sources of funding for the studies included in the review?

Q11^*^: If meta-analysis was performed, did the review authors use appropriate methods for statistical combination of results?

Q12: If meta-analysis was performed, did the review authors assess the potential impact of RoB in individual studies on the results of the meta-analysis or other evidence synthesis?

Q13^*^: Did the review authors account for RoB in individual studies when interpreting/discussing the results of the review?

Q14: Did the review authors provide a satisfactory explanation for, and discussion of, any heterogeneity observed in the results of the review?

Q15^*^: If they performed quantitative synthesis, did the review authors carry out an adequate investigation of publication bias (small study bias) and discuss its likely impact on the results of the review?

Q16: Did the review authors report any potential sources of conflict of interest, including any funding they received for conducting the review?

### 3.4. Reporting quality of included SRs

The PRISMA-2020 checklist contains 42 items, with a maximum score of 42 points. Based on the scores, two SRs (34, 36) were relatively complete and rated as high quality, two SRs (33, 42) had certain defects and were rated as medium quality, and the remaining nine SRs (31, 32, 35, 37–41, 43) had serious defects and were rated as low quality. In the case of the titles, nine SRs (31, 33, 34, 36, 39–43) fulfilled the criteria. None of the SRs completely reported all elements of the abstracts. The introduction section was comprehensive in all SRs. In the methods, two SRs (36, 41) had more comprehensive search details, and six SRs (31, 37, 39–41, 43) did not describe data processing for pooling and analyses. Finally, only one SR (36) provided a list of excluded studies in the results and four SRs (33, 34, 36, 42) evaluated outcome heterogeneity sources and bias risks. In the discussion section, all SRs (31–43) reported research limitations to varying degrees. Altogether, only two SRs (34, 36) had relatively completed reporting across all checklist items. The included SRs were generally of low reporting quality primarily due to methodological limitations in reporting, especially regarding data processing and presentation. Furthermore, the reporting on additional information, such as funding, conflicts of interest, and data access, was not transparent and had major inadequacies. The specific scores are shown in Table 4, and the categorization of reporting quality is shown in Table 5.

**Table 4:** Results of the PRISMA-2020 checklist.

**Item**	**Zhang and Hu (31)**	**Kang et al. (32)**	**Dennis et al. (33)**	**Zhu et al. (34)**	**Sang (35)**	**Wieland et al. (36)**	**Douglas et al. (37)**	**Susan and Beggs (38)**	**Manoj and Haider (39)**	**Alison et al. (40)**	**Christopher (41)**	**Holger et al. (42)**	**Paul and Ernst (43)**
Item 1	1	0	1	1	0	1	0	0	1	1	1	1	1
Item 2	0.5	0.5	0.5	0.5	0.5	0.5	0.5	0.5	0.5	0.5	0.5	0.5	0.5
Item 3	0.5	0.5	1	1	1	1	1	1	1	1	1	1	1
Item 4	0.5	1	1	1	1	1	1	1	1	1	1	1	1
Item 5	0.5	0.5	0.5	0.5	0.5	0.5	1	0.5	0.5	0.5	0.5	0.5	0.5
Item 6	0.5	0.5	0.5	0.5	0.5	1	0.5	0.5	0.5	0.5	1	0.5	0.5
Item 7	0	0	0	0	0	1	0	0	0	0	1	0	0
Item 8	0.5	0.5	0.5	1	0	1	0	0	0	0.5	0	0.5	0
Item 9	0	0	0.5	1	0	1	0	0	0	0	0	0.5	0.5
Item 10a	0	0	0	1	0	1	0	0	0	1	0	0	0
Item 10b	0.5	1	1	1	1	0.5	0	1	0	1	0	1	0
Item 11	0.5	0.5	1	1	0.5	1	0	0	0	1	0	1	0.5
Item 12	0	1	1	1	1	1	0	1	0	1	0	1	0
Item 13a	0	0	1	0	0	1	0	0	0	0	0	1	0
Item 13b	0	0	1	1	0	1	0	0	0	0	0	1	0
Item 13c	0	0	1	1	0	1	0	0	0	0	0	1	0
Item 13d	1	1	1	1	1	1	0	1	0	0	0	1	1
Item 13e	0	1	1	1	1	1	0	1	0	0	0	1	0
Item 13f	0	0	1	1	0	1	0	0	0	0	0	1	0
Item 14	0	0	1	1	1	1	0	0	0	0	0	1	0
Item 15	0	0	0	1	0	1	0	1	0	0	0	0	1
Item 16a	1	1	1	1	1	1	1	1	1	1	1	1	1
Item 16b	0	0	0	0	0	1	0	0	0	0	0	0	0
Item 17	1	1	1	1	1	1	1	1	1	1	1	1	1
Item 18	1	1	0.5	1	1	1	0	0	0	0	0	1	0
Item 19	0	1	1	1	1	1	0	1	0	1	0	1	0
Item 20a	0.5	0.5	0.5	0.5	0.5	1	0.5	0.5	0	0.5	0.5	1	0.5
Item 20b	1	1	1	1	1	1	1	1	0	1	1	1	1
Item 20c	0	1	1	1	0	1	0	0	0	0	0	1	0
Item 20d	0	0	1	1	0	1	0	0	0	0	0	1	0
Item 21	0	0	1	1	1	1	0	0	0	0	0	1	0
Item 22	0	0	1	1	0	1	0	1	0	0	0	0	1
Item 23a	1	0	1	1	0	1	1	1	1	1	1	1	1
Item 23b	1	1	1	1	1	1	1	1	1	1	1	1	1
Item 23c	1	1	1	1	1	1	1	1	1	1	1	1	1
Item 23d	1	0	0	1	1	1	1	1	1	1	1	1	1
Item 24a	0	0	1	1	0	0	0	0	0	0	0	0	0
Item 24b	0	0	0	1	0	0	0	0	0	0	0	0	0
Item 24c	0	0	0	1	0	1	0	0	0	0	0	0	0
Item 25	0	0	1	1	0	1	1	0	1	0	0	0	1
Item 26	0	0	1	1	0	1	0	0	1	0	0	0	0
Item 27	0	0	0	0	0	1	0	0	0	0	0	0	0
Total score	14.5	16.5	28.5	36	18.5	38.5	12.5	18	12.5	16.5	13.5	28.5	17

Full conformity is recorded as 1 point, partial conformity is recorded as 0.5 point, and no conformity is recorded as 0 point. Items corresponding to the evaluation theme: item 1 for the title; item 2 for the abstract; items 3–4 for the introduction; items 5–15 for the method; items 16a−22 for the results; items 23a−27 for the discussion.

**Table 5:** Degree and number of literature reports.

**Degree of literature reporting**	**Number of volumes**	**SRs**
Relatively complete	2	(34, 36)
Certain defects	2	(33, 42)
Serious flaws	9	(31, 32, 35, 37–41, 43)

Completeness of 80% or more (33–42 points) is considered “relatively complete” and high quality; completeness of 60% or more (25–32 points) is considered “certain defects” and medium quality; completeness of <60% (<25 points) is considered a “relatively serious lack of information” and low quality.

### 3.5. Evidence quality classification using GRADE

The pooled results of seven SRs (32–36, 38, 42) on the efficacy of yoga for CLBP regarding 4 outcomes, including pain, disability function, quality of life, and adverse events, were presented. The quality of evidence for the 92 outcomes was evaluated using GRADE. Moderate-quality evidence was obtained for 30 of the 92 outcomes (32.6%), low-quality evidence for 42 of 92 (45.7%), and very low-quality evidence for 20 of 92 (21.7%). Serious flaws in randomization, concealment, and blinding methods of the RCTs contained in the included literature were the main factors behind the downgrade. Other downgrading factors such as imprecision, publication bias, and inconsistency negatively affected the strength of the evidence. Further details are presented in Tables 6–**9**.

**Table 6:** Quality of evidence on pain relief with GRADE.

**Outcomes**	**Systematic review**	**Interventions vs. comparisons**	***N/n***	**MD (95%CI)**	***I* ^2^**	**Risk of bias**	**Inconsistency**	**Indirection**	**Imprecision**	**Publication bias**	**Quality of evidence**
**Pain**											
Pain at short term (1 week)	Wieland et al. (36)	Yoga vs. exercise	1/80	MD = −14.50, 95% CI = −22.92 to −6.08	None	−1	0	0	−1	0	⊕⊕°°^a^, ^c^
Pain at short term (4–6 weeks)	Dennis et al. (33)	Yoga vs. passive control	15/1,311	MD = −0.74; 95% CI = −1.04 to −0.44	*I*^2^ = 34%	−1	0	0	0	0	⊕⊕⊕°^a^
		Yoga vs. active control	10/1,167	MD = −0.78; 95% CI = −1.62 to 0.06	*I*^2^ = 80%	−1	−1	0	−1	−1	⊕°°°^a^, ^b^, ^c^, ^d^
	Zhu et al. (34)	Yoga vs. non-exercise	6/381	MD = −0.83, 95% CI −1.19 to −0.48	*I*^2^ = 0%	−1	0	0	−1	0	⊕⊕°°^a^, ^c^
		Yoga vs. physical exercise	5/350	MD = −0.37, 95% CI = −1.16 to 0.42	*I*^2^ = 81%	−1	−1	0	−1	0	⊕°°°^a^, ^b^, ^c^
	Wieland et al. (36)	Yoga vs. non-exercise	5/258	MD = −11.05, 95% CI = −14.22 to −7.88	*I*^2^ = 0%	−1	0	0	−1	0	⊕⊕°°^a^, ^c^
		Yoga vs. exercises	3/201	MD = −12.47, 95% CI = −18.28, −6.66	*I*^2^ = 36%	−1	0	0	−1	0	⊕⊕°°^a^, ^c^
	Holger et al. (42)	Yoga vs. control	6/584	SMD = −0.48; 95% CI = −0.65 to −0.31	*I*^2^ = 0%	−1	0	0	0	0	⊕⊕⊕°^a^
Pain at short-intermediate term (3–4 months)	Zhu et al. (34)	Yoga vs. non-exercise	10/1,031	MD = −0.43, 95% CI = −0.64 to −0.23	*I*^2^ = 0%	−1	0	0	0	0	⊕⊕⊕°^a^
		Yoga vs. physical exercise	4/564	MD = 0.19, 95% CI = −0.63 to 1.01	*I*^2^ = 64%	−1	−1	0	−1	0	⊕°°°^a^, ^b^, ^c^
	Wieland et al. (36)	Yoga vs. non-exercise	9/946	MD = −4.53, 95% CI = −6.61 to– 2.46	*I*^2^ = 0%	−1	0	0	0	0	⊕⊕⊕°^a^
		Yoga vs. exercise	2/326	MD = 2.68, 95%CI = −2.01 to 7.36	None	−1	0	0	−1	0	⊕⊕°°^a^, ^c^
		Yoga plus exercise vs. exercise	1/24	MD = −3.20, 95%CI = −13.76 to 7.36	None	−1	0	0	−1	0	⊕⊕°°^a^, ^c^
Pain at intermediate term (6 months)	Zhu et al. (34)	Yoga vs. non-exercise	8/823	MD = −0.56, 95% CI = −1.02 to −0.11	*I*^2^ = 50%	−1	−1	0	0	0	⊕⊕°°^a^, ^b^
		Yoga vs. physical exercise	4/392	MD = −0.73, 95% CI = −2.13 to 0.67	*I*^2^ = 85%	−1	−1	0	−1	0	⊕°°°^a^, ^b^, ^c^
	Wieland et al. (36)	Yoga vs. non-exercise	9/940	MD = −5.40, 95% CI = −8.58 to −2.22	*I*^2^ = 40%	−1	0	0	0	0	⊕⊕⊕°^a^
		Yoga vs. exercise	3/331	MD = −6.41, 95%CI = −21.66 to 8.83	*I*^2^ = 93%	−1	−1	0	−1	0	⊕°°°^a^, ^b^, ^c^
Pain at long term (12 months)	Dennis et al. (33)	Yoga vs. passive control	10/1,146	MD = −0.58; 95% CI = −0.94 to 0.22	*I*^2^ = 33%	−1	0	0	0	0	⊕⊕⊕°^a^
		Yoga vs. active control	5/663	MD = −0.62; 95% CI = −3.10 to 1.86	*I*^2^ = 91%	−1	−1	0	−1	0	⊕°°°^a^, ^b^, ^c^
	Zhu et al. (34)	Yoga vs. non-exercise	2/355	MD = −0.52, 95% CI = −1.64 to 0.59	*I*^2^ = 87%	−1	−1	0	−1	0	⊕°°°^a^, ^b^, ^c^
	Wieland et al. (36)	Yoga vs. non-exercise	3/521	MD = −5.87, 95% CI = −12.25 to 0.50	*I*^2^ = 68%	−1	−1	0	−1	0	⊕°°°^a^, ^b^, ^c^
	Wieland et al. (36)	Yoga vs. exercise	1/199	MD = 3.00, 95% CI = −4.25 to 10.25	None	−1	0	0	−1	0	⊕⊕°°^a^, ^c^
	Holger et al. (42)	Yoga vs. control	5/564	SMD = – 0.33; 95% CI = −0.59 to −0.07	*I*^2^ = 48%	−1	0	0	0	0	⊕⊕⊕°^a^
Pain at no staging	Sang (35)	Yoga vs. control	6/522	SMD = −0.41, 95% CI = −0.58 to−0.23	*I*^2^ = 0%	−1	0	0	0	0	⊕⊕⊕°^a^
	Susan and Beggs (38)	Yoga vs. control	5/381	*d* = 0.623, 95% CI = 0.377 to 0.868;	*I*^2^ = 22%	−1	0	0	−1	0	⊕⊕°°^a^, ^c^

Evidence quality: ⊕°°°, very low; ⊕⊕°°, low; ⊕⊕⊕°, moderate; ⊕⊕⊕⊕, high.

a Downgraded for limitations: studies with methodological flaws of blinding and allocation concealment.

b Downgraded for inconsistency: significant heterogeneity.

c Downgraded for imprecision: small-sample size, or wide confidence interval.

d Downgraded for publication bias:asymmetric funnel plots.

N/n, number of studies/number of participants; CI, confidence interval; I^2^, I-squared; d, effect size across the number of studies; MD, mean difference; SMD, standardized mean difference.

### 3.6. Efficacy evaluation

#### 3.6.1. Effects of yoga on pain relief

MAs were performed on six SRs (33–36, 38, 42) on the effect of yoga in relieving pain in cases of CLBP. A total of 25 pieces of evidence were obtained, including eight moderate, nine low, and eight very low-quality pieces of evidence. Pain was primarily measured using the visual analog scale (VAS). Out of the six studies, three SRs (33, 34, 36) with moderate-quality evidence showed that yoga considerably reduced pain compared with that reduced by non-exercise and passive controls [mean difference (MD) = −0.74, 95% confidence interval (CI): −1.04 to −0.44; MD = −0.43, 95% CI: −0.64 to −0.23; MD = −11.05, 95% CI = −14.22 to −7.88]. One SR (33) considered exercise and passive controls separately, showing no significant differences when comparing yoga intervention with the exercise control (MD = −0.78; 95% CI = −1.62 to 0.06; GRADE: very low) but showing a significant difference when comparing yoga intervention with the passive control (MD = −0.74; 95% CI = −1.04 to −0.44; GRADE: moderate). However, this difference only exists in the short term, as evidenced by a 12-month follow-up with no significant difference observed (MD = −0.58; 95% CI = −0.94 to 0.22; GRADE: moderate). Additionally, two SRs (34, 36) showed unclear results regarding whether yoga was more effective than non-exercise controls in the context of long-term efficacy at 12 months, with MD = −0.52, 95% CI = −1.64 to 0.59 (GRADE: very low) and MD = −5.87, 95% CI = −12.25 to 0.50 (GRADE: very low), respectively. Further details are presented in Table 6.

#### 3.6.2. Effects of yoga on disability or back-specific functions

MAs were performed on six SRs (32–34, 36, 38, 42) on the effects of yoga on disability or back-specific functions, which were analyzed using the Oswestry Disability Index or Roland Morris Disability Questionnaire. A total of 25 pieces of evidence were obtained, comprising 11 moderate-, 13 low-, and one very low-quality pieces of evidence. One SR (34) compared the efficacy of yoga and physical exercise for improving functional disability associated with low back pain, and the results showed no statistically significant differences between the efficacy of yoga and physical therapy, with the quality of evidence ranging from moderate to very low. This finding indicated that the efficacy of yoga was not significantly greater than that of physical therapy for improving lumbar functional disability. One SR (36) examined the effect of yoga on back-specific function. Five pieces of evidence showed no significant difference between the efficacy of yoga and conventional exercise (GRADE: from moderate to low). Seven pieces of evidence from two studies (34, 36) suggest that yoga can improve back function and disability compared with non-exercise, and further details are presented in Table 7.

**Table 7:** Quality of evidence on disability or back-specific function with GRADE.

**Outcomes**	**Systematic review**	**Interventions vs. comparisons**	***N/n***	**MD (95% CI)**	***I* ^2^**	**Risk of bias**	**Inconsistency**	**Indirection**	**Imprecision**	**Publication bias**	**Quality of evidence**
**Disability**											
Disability at short term (4–6 weeks)	Dennis et al. (33)	Yoga vs. passive control	15/1,327	MD = −2.28, 95% CI = −3.30 to −1.26	*I*^2^ = 38%	−1	0	0	0	0	⊕⊕⊕°^a^
		Yoga vs. active control	10/1,179	MD = −2.04, 95% CI = −4.02 to −0.06	*I*^2^ = 77%	−1	−1	0	0	0	⊕⊕°°^a, b^
	Zhu et al. (34)	Yoga vs. non-exercise	7/397	SMD = −0.30, 95% CI = −0.51 to−0.10	*I*^2^ = 0%	−1	0	0	0	0	⊕⊕⊕°^a^
		Yoga vs. physical exercise	3/272	MD = −0.34, 95%CI = −1.60 to 0.92	*I*^2^ = 0%	−1	0	0	−1	0	⊕⊕°°^a, c^
	Holger et al. (42)	Yoga vs. control	8/689	SMD = – 0.59, 95% CI = −0.87 to −0.30	*I*^2^ = 59%	−1	−1	0	0	0	⊕⊕°°^a, b^
Disability at short-intermediate term (3–4 months)	Zhu et al. (34)	Yoga vs. non-exercise	9/951	SMD = −0.31, 95% CI = −0.45 to −0.18	*I*^2^ = 30%	−1	0	0	0	0	⊕⊕⊕°^a^
		Yoga vs. physical exercise	4/519	MD = −0.04, 95% CI = −1.76 to 1.67	*I*^2 =^ 67%	−1	−1	0	−1	0	⊕°°°^a, b, c^
Disability at intermediate term (6 months)	Zhu et al. (34)	Yoga vs. non-exercise	6/688	SMD = −0.38, 95% CI = −0.53 to −0.23	*I*^2 =^ 0%	−1	0	0	0	0	⊕⊕⊕°^a^
		Yoga vs. physical exercise	2/229	MD = −1.32, 95% CI = −2.78 to 0.13	*I*^2^ = 0%	−1	0	0	−1	0	⊕⊕°°^a, c^
Disability at long term (12 months)	Dennis et al. (33)	Yoga vs. passive control	11/1,225	MD = −2.34, 95% CI = −3.30 to −1.38	*I*^2^ = 27%	−1	0	0	0	0	⊕⊕⊕°^a^
		Yoga vs. active control	5/675	MD = −2.04, 95% CI = −4.02 to −0.06	*I*^2^ = 77%	−1	−1	0	0	0	⊕⊕°°^a, b^
	Zhu et al. (34)	Yoga vs. non-exercise	2/365	SMD = −0.33, 95% CI = −0.54 to −0.12	*I*^2^ = 9%	−1	0	0	−1	0	⊕⊕°°^a, c^
	Holger et al. (42)	Yoga vs. control	5/574	SMD = – 0.35, 95% CI = −0.55 to −0.15	*I*^2^ = 20%	−1	0	0	0	0	⊕⊕⊕°^a^
Disability at no staging	Kang et al. (32)	Yoga vs. routine group	14/1,684	MD = −1.86, 95%CI = −2.39 to −1.33	*I*^2^ = 17%	−1	0	0	0	−1	⊕⊕°°^a, d^
	Susan and Beggs (38)	Yoga vs. control	8/743	*d* = 0.645, 95% CI = 0.496 to 0.795	*I*^2^ = 0%	−1	0	0	0	0	⊕⊕⊕°^a^
**Back-specific function**											
At very short term (1 week)	Wieland et al. (36)	Yoga vs. exercise	1/80	MD = −1.25, 95%CI = −1.73 to −0.77	None	−1	0	0	−1	0	⊕⊕°°^a, c^
At short term (4 to 8 weeks)	Wieland et al. (36)	Yoga vs. non-exercise	8/474	MD = −0.41, 95% CI = −0.61 to −0.21	*I*^2^ = 6%	−1	0	0	0	0	⊕⊕⊕°^a^
		Yoga vs. exercise	4/395	MD = −0.04, 95%CI = −0.32 to 0.23	*I*^2^ = 42%	−1	0	0	−1	0	⊕⊕°°^a, c^
At short-intermediate term (3 months)	Wieland et al. (36)	Yoga vs. non-exercise	11/1,155	MD = −0.31, 95% CI = −0.50 to −0.12	*I*^2^ = 55%	−1	−1	0	0	0	⊕⊕°°^a, b^
		Yoga vs. exercise	4/575	MD = −0.08, 95%CI = −0.28to 0.13	*I*^2^ = 31%	−1	0	0	0	0	⊕⊕⊕°^a^
		Yoga plus exercise vs. exercise	1/24	MD = −3.68, 95%CI = −8.44, 1.08	None	−1	0	0	−1	0	⊕⊕°°^a, c^
At intermediate term (6 months)	Wieland et al. (36)	Yoga vs. non-exercise	11/1,157	MD = −0.36, 95% CI = −0.52 to −0.21	*I*^2^ = 38%	−1	0	0	0	0	⊕⊕⊕°^a^
		Yoga vs. exercise	3/333	MD = −0.08, 95%CI = −0.40 to 0.23	*I*^2^ = 47%	−1	0	0	−1	0	⊕⊕°°^a, c^
At long term (12 months)	Wieland et al. (36)	Yoga vs. non-exercise	3/532	MD = −0.27, 95% CI = −0.45 to −0.10	*I*^2^ = 0%	−1	0	0	0	0	⊕⊕⊕°^a^
		Yoga vs. exercise	1/200	MD = −0.02, 95% CI = −0.29 to 0.26	None	−1	0	0	−1	0	⊕⊕°°^a, c^

Evidence quality: ⊕°°°, very low; ⊕⊕°°, low; ⊕⊕⊕°, moderate; ⊕⊕⊕⊕, high.

a Downgraded for limitations: studies with methodological flaws of blinding and allocation concealment.

b Downgraded for inconsistency: significant heterogeneity.

c Downgraded for imprecision: small-sample size, or wide confidence interval.

d Downgraded for publication bias: asymmetric funnel plots.

N/n, number of studies/number of participants; CI, confidence interval; I^2^, I-squared; d, effect size across the number of studies; MD, mean difference; SMD, standardized mean difference.

#### 3.6.3. Effects of yoga on the quality of life

Four SRs (33, 34, 36, 42) analyzed the effect of yoga on the quality of life of patients with CLBP, generating a total of 38 pieces of evidence, which comprised 11 moderate-, 17 low-, and 10 very low-quality pieces of evidence. Factors such as physical and mental health were included in the assessment of the quality of life. One SR (42) showed that compared with controls, yoga improved the quality of life in the short and long terms. Furthermore, three SRs (33, 34, 36) showed that compared with no exercise, yoga positively affected physical function and mental health in the short- to long-term course of pain. However, most pieces of evidence showed no statistically significant differences (with the certainty of evidence ranging from moderate to very low) and showed large CIs. Thus, the efficacy of yoga in improving the physical and psychological quality of life remains unclear. Further details are presented in Table 8.

**Table 8:** Quality of evidence on the quality of life with GRADE.

**Outcomes**	**Systematic review**	**Interventions vs. comparisons**	***N/n***	**MD (95% CI)**	***I* ^2^**	**Risk of bias**	**Inconsistency**	**Indirection**	**Imprecision**	**Publication bias**	**Quality of evidence**
**Physical quality of life**											
At short term	Zhu et al. (34)	Yoga vs. non-exercise	1/13	MD = 0.75, 95% CI = −11.45 to 12.95	None	−1	−1	0	−1	0	⊕°°°^a, b, c^
	Dennis et al. (33)	Yoga vs. passive control	9/980	MD = 2.80, 95% CI = 1.00 to 4.70	*I*^2^ = 24%	−1	0	0	0	0	⊕⊕⊕°^a^
		Yoga vs. active control	8/1,039	MD = 5.10, 95% CI = −0.30 to 10.50	*I*^2^ = 88%	−1	−1	0	−1	0	⊕°°°^a, b, c^
	Wieland et al. (36)	Yoga vs. non-exercise	2/81	MD = 0.50, 95% CI = 0.05 to 0.95	*I*^2^ = 0%	−1	0	0	−1	0	⊕⊕°°^a, c^
		Yoga vs. exercise	3/219	MD = 1.03, 95% CI = 0.36 to 1.71	*I*^2^ = 82%	−1	−1	0	−1	0	⊕°°°^a, b, c^
At short to intermediate term	Zhu et al. (34)	Yoga vs. non-exercise	5/617	SMD = 0.06, 95% CI = −0.10 to 0.22	*I*^2^ = 0%	−1	0	0	0	0	⊕⊕⊕°^a^
		Yoga vs. physical exercise	2/348	MD = 0.18, 95% CI = −1.97 to 2.32	*I*^2^ = 0%	−1	0	0	0	0	⊕⊕⊕°^a^
	Wieland et al. (36)	Yoga vs. non-exercise	6/686	MD = 0.20, 95% CI = 0.03 to 0.37	*I*^2^ = 10%	−1	0	0	0	0	⊕⊕⊕°^a^
		Yoga vs. exercise	1/237	MD = 0.15, 95% CI = −0.11to 0.40	None	−1	0	0	−1	0	⊕⊕°°^a, c^
At intermediate term	Zhu et al. (34)	Yoga vs. non-exercise	2/366	SMD = 0.08, 95% CI = −0.13 to 0.28	*I*^2^ = 0%	−1	0	0	0	0	⊕⊕⊕°^a^
		Yoga vs. physical exercise	1/107	MD = −0.34, 95% CI = −12.77 to 12.09	None	−1	0	0	−1	0	⊕⊕°°^a, c^
	Wieland et al. (36)	Yoga vs. non-exercise	3/434	MD = 0.16, 95% CI = −0.13 to 0.46	*I*^2^ = 52%	−1	−1	0	0	0	⊕⊕°°^a, b^
		Yoga vs. exercise	1/54	MD = 1.34, 95% CI = 0.75 to 1.94	None	−1	0	0	−1	0	⊕⊕°°^a, c^
At long term	Zhu et al. (34)	Yoga vs. non-exercise	1/264	MD = 0.79, 95% CI = −1.52 to 3.10	None	−1	0	0	−1	0	⊕⊕°°^a, c^
	Dennis et al. (33)	Yoga vs. passive control	6/725	MD = 2.20, 95% CI = 0.30 to 4.10	*I*^2^ = 0%	−1	0	0	0	0	⊕⊕⊕°^a^
		Yoga vs. active control	3/283	MD = 3.10, 95% CI = −19.50 to 25.60	*I*^2^ = 93%	−1	−1	0	−1	0	⊕°°°^a, b, c^
	Wieland et al. (36)	Yoga vs. non-exercise	1/264	MD = 0.17, 95% CI = −0.07 to 0.41	None	−1	0	0	−1	0	⊕⊕°°^a, c^
		Yoga vs. exercise	1/80	MD = 1.06, 95% CI = 0.59 to 1.53	None	−1	0	0	−1	0	⊕⊕°°^a, c^
**Mental quality of life**											
At short term	Dennis et al. (33)	Yoga vs. passive control	7/845	MD = 1.70, 95% CI = 0.20 to 3.20	*I*^2^ = 0%	−1	0	0	0	0	⊕⊕⊕°^a^
		Yoga vs. active control	7/929	MD = 5.70, 95% CI = −2.50 to 14.00	*I*^2^ = 92%	−1	−1	0	−1	0	⊕°°°^a, b, c^
	Zhu et al. (34)	Yoga vs. non-exercise	1/13	MD = −4.71, 95% CI = −21.66 to 12.24	None	−1	−1	0	−1	0	⊕°°°^a, b, c^
	Wieland et al. (36)	Yoga vs. non-exercise	2/81	MD = −0.15, 95% CI = −1.24 to 0.93	*I*^2^ = 67%	−1	−1	0	−1	0	⊕°°°^a, b, c^
		Yoga vs. exercise	3/219	MD = 1.03, 95% CI = −0.44 to 2.51	*I*^2^ = 96%	−1	−1	0	−1	0	⊕°°°^a, b, c^
At short to intermediate term	Zhu et al. (34)	Yoga vs. non-exercise	5/617	SMD = 0.15, 95% CI = −0.01 to 0.31	*I*^2^ = 0%	−1	0	0	0	0	⊕⊕⊕°^a^
		Yoga vs. physical exercise	2/348	MD = 0.07, 95% CI = −2.74 to 2.89	*I*^2^ = 0%	−1	0	0	0	0	⊕⊕⊕°^a^
	Wieland et al. (36)	Yoga vs. non-exercise	6/686	MD = 0.20, 95% CI = 0.05 to 0.35	*I*^2^ = 0%	−1	0	0	0	0	⊕⊕°°^a, c^
		Yoga vs. exercise	1/237	MD = 0.16, 95% CI = −0.10 to 0.41	None	−1	0	0	−1	0	⊕⊕°°^a, c^
At intermediate term	Zhu et al. (34)	Yoga vs. non-exercise	2/366	SMD = 0.18, 95% CI = −0.03 to −0.39	*I*^2^ = 0%	−1	0	0	0	0	⊕⊕⊕°^a^
		Yoga vs. physical exercise	1/107	MD = 1.53, 95% CI = −6.43 to −9.49	None	−1	0	0	−1	0	⊕⊕°°^a, c^
	Wieland et al. (36)	Yoga vs. non-exercise	3/434	MD = 0.21, 95% CI = 0.00 to 0.41	*I*^2^ = 9%	−1	0	0	0	0	⊕⊕⊕°^a^
		Yoga vs. exercise	1/54	MD = 1.33, 95% CI = 0.74 to1.92	None	−1	0	0	−1	0	⊕⊕°°^a, c^
At long term	Dennis et al. (33)	Yoga vs. passive control	4/595	MD = 1.30, 95% CI = −2.30 to 4.80	*I*^2^ = 39%	−1	0	0	−1	0	⊕⊕°°^a, c^
		Yoga vs. active control	2/173	MD = 6.40, 95% CI = −78.10 to 91.00	*I*^2^ = 93%	−1	−1	0	−1	0	⊕°°°^a, b, c^
	Zhu et al. (34)	Yoga vs. non-exercise	1/264	MD = 0.42, 95% CI = −2.16 to 3.00	None	−1	0	0	−1	0	⊕⊕°°^a, c^
	Wieland et al. (36)	Yoga vs. non-exercise	1/264	MD = 0.07, 95% CI = −0.17 to 0.31	None	−1	0	0	−1	0	⊕⊕°°^a, c^
		Yoga vs. exercise	1/80	MD = 0.87, 95% CI = 0.41 to 1.33	None	−1	0	0	−1	0	⊕⊕°°^a, c^
**Quality of life**											
At short term	Holger et al. (42)	Yoga vs. control	4/388	SMD = 0.41; 95% CI = 0.11 to 0.93	*I*^2^ = 72%	−1	−1	0	−1	0	⊕°°°^a, b, c^
At long term	Holger et al. (42)	Yoga vs. control	2/287	SMD = 0.18; 95% CI = 0.05 to 0.41	*I*^2^ = 0%	−1	0	0	−1	0	⊕⊕°°^a, c^

Evidence quality: ⊕°°°, very low; ⊕⊕°°, low; ⊕⊕⊕°, moderate; ⊕⊕⊕⊕, high.

a Downgraded for limitations: studies with methodological flaws of blinding and allocation concealment.

b Downgraded for inconsistency: significant heterogeneity.

c Downgraded for imprecision: small-sample size, or wide confidence interval.

N/n, number of studies/number of participants; CI, confidence interval; I^2^, I-squared; MD, mean difference; SMD, standardized mean difference.

### 3.7. Safety of yoga for low back pain

Six SRs (33, 34, 36, 37, 42, 43) showed adverse events associated with yoga for treating low back pain. Most adverse events were the mild-to-moderate exacerbation of low back pain. More severe adverse events included herniated disks and intense pain. MAs were performed on two SRs (33, 36) that focused on these adverse events. One SR (33) showed no significant difference in the incidence of adverse events between the yoga and active control groups [RR (risk ratio) = 0.58; 95% CI = 0.28–1.19; GRADE: low]. The other SR (36) showed that yoga and conventional exercise exhibited comparable safety profiles. Overall, yoga was not associated with serious adverse events; however, more studies are warranted to further investigate the safety of these interventions. Additional details are presented in Table 9.

**Table 9:** Quality of evidence on adverse events with GRADE.

**Outcomes**	**Systematic review**	**Interventions vs. comparisons**	***N/n***	**MD (95% CI)**	***I* ^2^**	**Risk of bias**	**Inconsistency**	**Indirection**	**Imprecision**	**Publication bias**	**Quality of evidence**
Adverse events	Dennis et al. (33)	Yoga vs. passive control	9/949	RR = 3.78; 95% CI = 1.79 to 7.98	*I*^2^ = 0%	−1	0	0	0	0	⊕⊕⊕°^a^
		Yoga vs. active control	7/775	RR = 0.58; 95% CI = 0.28 to 1.19	*I*^2^ = 69%	−1	−1	0	0	0	⊕⊕°°^a, b^
	Wieland et al. (36)	Yoga vs. non-exercise	8/1,037	RR = 4.76; 95% CI = 2.08 to 10.89	*I*^2^ = 0%	−1	0	0	0	0	⊕⊕⊕°^a^
		Yoga vs. exercise	5/6,40	RR = 0.93; 95% CI = 0.56 to 1.53	*I*^2^ = 0%	−1	0	0	0	0	⊕⊕⊕°^a^

Evidence quality: ⊕°°°, very low; ⊕⊕°°, low; ⊕⊕⊕°, moderate; ⊕⊕⊕⊕, high.

a Downgraded for limitations: studies with methodological flaws of blinding and allocation concealment.

b Downgraded for inconsistency: significant heterogeneity.

N/n, number of studies/number of participants; CI, confidence interval; I^2^, I-squared; RR, risk ratio.

### 3.8. Overlap

**Figure 2:**
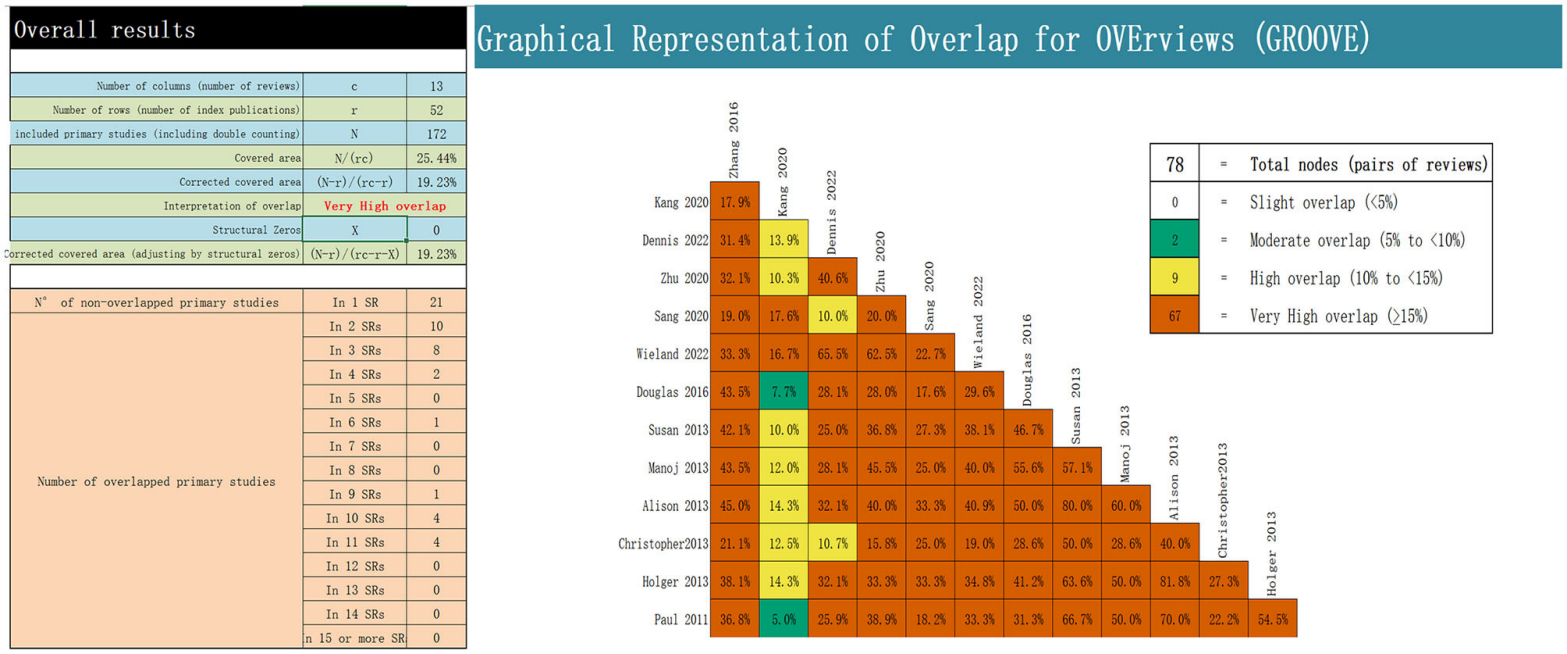
Overlapping of the included reviews.

Graphical representation of overlap for overviews computes the overall CCA and provides a new graphical representation of the overlap between each pair of possible SRs/MAs. A total of 13 SRs comprised 172 RCTs. Of these, 52 RCTs overlapped, showing a calculated CCA of 19.23%. A total of 78 nodes between the reviews were observed, of which two were moderately overlapping, nine were highly overlapping, and 67 were very highly overlapping. Further details are presented in Figure 2.

## 4. Discussion

The vast majority of patients with CLBP, a common MSK, complain of lumbosacral pain that lasts for an extended period. Moreover, the onset of the disease is insidious and not easily noticed by patients; thus, the disease puts a great burden on the mental and financial conditions of patients. The way a clinician approaches a patient with CBLP profoundly affects CLBP treatment because the patient may specifically remember negative inhibitory information and develop avoidance behavior and panic beliefs (21). Owing to the difficulty in determining CLBP pathogenesis, a clear pathological basis is unavailable for clinically available laboratory measures, and the subjective analysis of pain caused by the disease is common. Therefore, in the context of the modern biopsychosocial model of medicine, the American College of Physicians Guidelines for CLBP recommend a combination of physical and psychological treatments (12), and yoga meets this need.

Yoga, which originated in ancient India, is a form of physical and mental exercise that comprises meditative relaxation, breathing, and asanas. Meditation practice helps focus the mind and allows the practitioner to direct their awareness to breathing. Moreover, modern scientific research has shown that meditation can increase the levels of neurotransmitters, including melatonin and gamma-aminobutyric acid, and endorphins, thus playing a positive role in reducing mental stress and its effects in humans (44). Additionally, yoga asana practice can improve muscle strength, joint flexibility, and balance in patients with CLBP. Tilbrook et al. (45) found that after a 12-week yoga program, the back muscle function of adults with CLBP improved over 12 months, suggesting that yoga strengthened muscle stability and reduced low back pain by increasing hip and spinal flexibility. Thus, we searched for the published literature related to the SR of yoga in CLBP treatment to evaluate the effectiveness of yoga for managing CLBP and verify whether it is a supplementary and alternative treatment strategy for patients with CLBP.

We systematically evaluated the included studies using the AMSTAR-2 scale, PRISMA-2020 statement, and GRADE system. In terms of methodology, most critical factors were reported poorly because most studies lacked a presentation and description of the used preliminary research protocol and literature search, excluded reference lists, and failed to address sources of heterogeneity and risk of bias such as publication bias. Emphasizing the abovementioned factors can improve the methodological quality of SRs/MAs. In terms of reporting quality, the included SRs were generally of low reporting quality, primarily because of methodological flaws in reporting, especially regarding data processing and presentation. In terms of evidence quality, most SRs were characterized by inadequate or unreasonable randomization, blinding, and allocation concealment. Simultaneously, a significant risk of heterogeneity and homogeneity, mainly related to the small number of trials and the number of participants, as well as indicators observed in a single body of evidence, was present. Additionally, the repetition rate of the original RCT results extracted using the GROVE tool was very high, which might have resulted in some bias. Therefore, the careful handling and discussion of all aspects of a research design and its implementation are necessary. Moreover, higher-quality, large-sample, multi-center RCTs should be conducted to improve the homogeneity of evidence sources (46).

The present study showed that yoga exerted a certain therapeutic effect to improve pain and functional disability; however, the low-quality results reduced the credibility of the evidence. With respect to pain scores, most evidence pieces supported the efficacy of yoga in managing CLBP. Compared with non-exercise measures, yoga interventions significantly improved pain scores in patients with low back pain. Wieland et al. (36) showed a large pooled effect for yoga compared with non-exercise measures on pain scores, especially in the short term of 4–6 weeks (MD = −11.05, 95% CI = −14.22 to −7.88). However, this effect gradually decreased after 3 months (MD = −4.53, 95% CI = −6.61 to −2.46). Zhu et al. (34) showed no statistical significance regarding the long-term efficacy of yoga (MD = −0.52, 95% CI = −1.64 to 0.59). These results indicate that yoga probably exhibits better short-term efficacy than a non-exercise measure. Additionally, we found that compared with physical and exercise therapies, yoga did not exert remarkable effects, even though many results were statistically significant. Moreover, many MD values were less than the clinical minimum important difference. In this comparison, the pain improvement due to yoga was not considerable. Dennis et al. (33) distinguished passive exercises from active exercises. Compared with active exercises, yoga did not show statistical significance (MD = −0.78; 95% CI = −1.62 to 0.06), whereas compared with passive exercises, yoga showed significant results (MD = −0.74; 95% CI = −1.04 to −0.44) for CLBP management. Nevertheless, this difference decreased in follow-ups longer than 12 months (no statistical significance), suggesting a minor long-term pain improvement with yoga. In terms of improving functional disabilities, the study showed that yoga was advantageous in improving functional disability, which was more pronounced in a follow-up study by Dennis et al. (33), and Kang et al. (32) also found a significant advantage. However, Zhu et al. (34) found that compared with physical therapy, yoga showed no statistically significant results, suggesting that yoga might not be advantageous over physical therapy. Because the mentioned results have been pooled from a small number of studies, publication bias cannot be ruled out. In terms of improving the quality of life, yoga may not be effective compared with other controls, as evidenced by many non-significant results. With respect to a short- or long-term improvement in the quality of life, most evidence pieces (GRADE evidence from very low to moderate) showed that yoga had no significant advantage. These findings suggest that yoga may not have a noticeable therapeutic effect on improving the quality of life of patients with low back pain. However, we think that this inference may also be related to publication bias because these results are pooled from small-sample studies, and large-sample RCTs have not been conducted yet. Thus, future studies should verify this inference. Next, two studies (33, 36) assessed the safety of yoga for patients with low back pain using adverse events as the outcome. Wieland et al. (36) found that yoga was associated with a significantly increased risk of adverse events compared to non-exercise controls (RR = 4.76; 95% CI = 2.08–10.89); however, no statistical difference was observed when yoga intervention was compared with other exercises. Dennis et al. (33) showed no statistical difference in the incidence of adverse events between yoga and other conventional exercise groups, consistent with the Wieland et al. results. Therefore, we believe that yoga has the same safety profile as other exercise therapies; thus, it can be recommended as a supplementary exercise therapy for treating low back pain.

### 4.1. Strengths and limitations

To the best of our knowledge, this is the first study to assess the quality of methodologies and evidence pieces gathered from studies on yoga for CLBP and provide a specific evidence-based medical basis for formulating clinical guidelines. However, the study has certain objective limitations. First, evaluating the quality of methodologies and evidence pieces is subjective. Even if we evaluated each item of the evaluation system in detail and objectively, the overall confidence of most SRs was low, which led to a considerable risk of bias and uncertainty. Second, although we drafted a plan (Appendix 2) before implementing this review, it was not officially registered on PROSPERO (International Prospective Registration for System Review), and a reporting bias may exist. Finally, as the main outcome indicator of interest is the effect of yoga on pain, further research is necessary to clarify the potential benefits of yoga in improving balance, reducing the risk of falls, and increasing musculoskeletal strength.

### 4.2. Implications

This SR offers implications at several levels as follows: (a) From the perspective of the evidence obtained and its systematic evaluation: similar to the results of the SRs, the overview by Roberta pointed out that yoga significantly improved pain, especially low back pain (47). Furthermore, the small-sample size and lack of appropriate methods reduced the quality of evidence, leading to unclarity regarding the benefits of yoga. Additionally, compared with recent clinical guidelines (12), which recommend incorporating yoga as a non-pharmacological treatment option for CLBP, the present findings support this recommendation by showing that yoga can help improve pain and dysfunction in patients and is a relatively safe physical and mental exercise. (b) From the perspective of the clinical treatment mode for patients experiencing pain: while treating chronic musculoskeletal pain, the concept of comprehensive guidance through a person-centered approach (including biological, psychological, and social factors) is crucial and determines the effectiveness of interventions; thus, finding low-cost treatment options to treat chronic non-specific pain will offer greater benefits to patients (48), and yoga is a proactive intervention method that meets the characteristics of low cost and high patient acceptance. Additionally, clinicians are recommended to appropriately shift their attention to biomechanics and anatomical pathology to humanistic factors such as the social psychology of patients. Thus, by creating a positive and autonomous medical and health environment, the avoidance behavior and panic beliefs of patients caused by negative inhibitory information can be avoided and active physical activity can be promoted (49).

## 5. Conclusion

In conclusion, yoga appears to be an effective and safe non-pharmacological strategy for treating CLBP. Currently, it may exhibit better efficacy in improving pain and functional disability associated with CLBP. However, owing to the generally low quality and certainty of the evidence pooled from the included SRs, the present results should be interpreted cautiously. In addition, we found numerous non-significant results and low-quality evidence regarding yoga practice to improve the quality of life in patients with CLBP. Therefore, whether yoga is different from other exercise or non-exercise therapies in improving the quality of life remains unclear. Nevertheless, the evidence presented herein is mostly obtained from small-sample studies without verification and validation from large-sample MAs.

## Data availability statement

The original contributions presented in the study are included in the article/Supplementary material, further inquiries can be directed to the corresponding authors.

## Author contributions

XZ: Conceptualization, Formal analysis, Methodology, Writing—original draft. TC: Conceptualization, Formal analysis, Methodology, Supervision, Writing—original draft. WH: Conceptualization, Data curation, Supervision, Validation. MS: Conceptualization, Formal analysis, Methodology, Supervision. YC: Conceptualization, Data curation, Supervision, Validation. SW: Data curation, Formal analysis. GZ: Data curation, Formal analysis, Methodology. MH: Data curation, Formal analysis, Methodology. MZ: Conceptualization, Data curation, Formal analysis. JY: Conceptualization, Data curation, Formal analysis. HY: Conceptualization, Data curation, Formal analysis. LZ: Data curation, Formal analysis. CZ: Data curation, Formal analysis. ZL: Funding acquisition, Writing—review & editing. XL: Funding acquisition, Writing—review & editing.
