# Supplementary material for: Efficacy and safety of yoga for the management of chronic low back pain: an overview of systematic reviews
Source: Front Neurol. 2023 Oct 27;14:1273473. doi: 10.3389/fneur.2023.1273473 (PMC10641484; doi:10.3389/fneur.2023.1273473)
Supplement: Supplementary file 1 [file Data_Sheet_1.docx]

**Appendix 1. Search strategies of each database**

The following database will be searched from inception to September 2023.

| **Database 1Pubmed** | |
| --- | --- |
| **Number** | **Search terms** |
| #1 | Yoga[MeSH Terms] |
| #2 | (yogic[Title/Abstract])) OR (yogi[Title/Abstract])) OR (yog*[Title/Abstract])) |
| #3 | #1 OR #2 |
| #4 | Low Back Pain[Mesh] |
| #5 | Low back pain[Title/Abstract]) OR (low back pains[Title/Abstract])) OR (lumbago[Title/Abstract])) OR (lower back pain[Title/Abstract])) OR (lower back pains[Title/Abstract])) OR (low back ache[Title/Abstract])) OR (low back aches[Title/Abstract])) OR (low backache[Title/Abstract])) OR (low backaches[Title/Abstract])) OR (lumbar pain[Title/Abstract])) OR (herniated disk[Title/Abstract])) OR (herniated disc[Title/Abstract])) OR (hernia intervertebral disc[Title/Abstract])) OR (lumbar degenerat*[Title/Abstract])) OR (backache[Title/Abstract])) OR (back disorders[Title/Abstract])) OR (sciatica[Title/Abstract])) OR (coccyx[Title/Abstract])) OR (coccy*[Title/Abstract])) OR (spondylosis[Title/Abstract]) |
| #6 | #4 OR #5 |
| #7 | "Systematic Review" [Publication Type]) OR ("Systematic Reviews as Topic"[Mesh])) OR ("Meta-Analysis" [Publication Type])) OR ("Meta-Analysis as Topic"[Mesh])) OR (Systematic review[Title/Abstract])) OR (Meta-analysis[Title/Abstract]) |
| #8 | #3 AND #6AND #7 |
| **Database 2 Cochrane library** | |
| **Number** | **Search terms** |
| #1 | MeSH descriptor: [Yoga] explode all trees |
| #2 | (yogic, OR yogi, OR yog*):ti,ab,kw |
| #3 | #1 OR #2 |
| #4 | MeSH descriptor: [Low Back Pain] explode all trees |
| #5 | (low back pain, OR low back pains, OR lumbago, OR lower back pain, OR lower back pains, OR low back ache, OR low back aches, OR low backache, OR low backaches, OR lumbar pain, OR herniated disk, OR herniated disc, OR hernia intervertebral disc, OR lumbar degenerat*, OR backache, OR back disorders, OR sciatica, OR coccyx, OR coccy*, OR spondylosis):ti,ab,kw |
| #6 | #4 OR #5 |
| #7 | MeSH descriptor: [systematic review] explode all trees OR MeSH descriptor: [systematic review as Topic] explode all trees |
| #8 | (systematic review OR Meta-analysis):ti,ab,kw |
| #9 | #7 OR #8 |
| #10 | #3 AND #6 AND #9 |
| **Database 3 EMBASE** | |
| **Number** | **Search terms** |
| #1 | 'yoga'/exp |
| #2 | 'yoga':ab,ti OR 'yogic':ab,ti OR 'yogi':ab,ti OR 'yog*':ab,ti |
| #3 | #1 OR #2 |
| #4 | 'low back pain'/exp |
| #5 | 'low back pain':ab,ti OR 'low back pains':ab,ti OR lumbago:ab,ti OR 'lower back pain':ab,ti OR 'lower back pains':ab,ti OR 'low back ache':ab,ti OR 'low back aches':ab,ti OR 'low backache':ab,ti OR 'low backaches':ab,ti OR 'lumbar pain':ab,ti OR 'herniated disk':ab,ti OR 'herniated disc':ab,ti OR 'hernia intervertebral disc':ab,ti OR 'lumbar degenerat*':ab,ti OR backache:ab,ti OR 'back disorders':ab,ti OR sciatica:ab,ti OR coccyx:ab,ti OR coccy*:ab,ti OR spondylosis:ab,ti |
| #6 | #4 OR #5 |
| #7 | 'systematic review'/exp OR 'systematic review (topic)'/exp OR'meta analysis'/exp OR 'meta analysis (topic)'/exp OR'systematic review':ab,ti OR'meta analysis':ab,ti |
| #8 | #3 AND #6AND #7 |
| **Database 4 Web of Science** | |
| **Number** | **Search terms** |
| #1 | TS=(Yoga OR yoga OR yogic OR yogi OR yog*) |
| #2 | TS=(Low Back Pain OR low back pain OR low back pains OR lumbago OR lower back pain OR lower back pains OR low back ache OR low back aches OR low backache OR low backaches OR lumbar pain OR herniated disk OR herniated disc OR hernia intervertebral disc OR lumbar degenerat* OR backache OR back disorders OR sciatica OR coccyx OR coccy* OR spondylosis) |
| #3 | TS=(Systematic Review OR Meta-analysis) |
| #4 | #1 AND #2 AND #3 |
|  | **Database 5 PEDro Database** |
| **Number** | **Search terms** |
| #1 | Abstract and Title: Yoga AND Low back pain |
| #2 | Method: Systematic Review |
| #3 | #1 AND #2 |
| **Database 6 China National Knowledge Infrastructure (CNKI)** | |
| (SU=瑜伽 AND SU=下腰痛+腰椎间盘突出症+腰背疼痛+腰椎盘突出症+腰椎盘突出+椎间盘突出+腰腿痛+下腰痛+下背痛+腰椎间盘纤维环破裂症+腰突症+椎间盘膨出+腰椎间盘膨出+腰椎椎间盘突出+下腰疼+腰椎间盘脱出症+腰痛+腰椎间盘脱出+腰背痛+腰间盘突出症+椎间盘突出症+腰椎间盘突出+腰间盘突出AND SU=系统评价+系统综述＋元分析＋荟萃分析＋meta) | |
| **Database 7 Wanfang Database** | |
| 主题:(“瑜伽”) AND (“下腰痛” OR “腰椎间盘突出症” OR “腰背疼痛” OR “腰椎盘突出症” OR “腰椎盘突出” OR “椎间盘突出”OR “腰腿痛” OR “下腰痛” OR “下背痛” OR “腰椎间盘纤维环破裂症” OR “腰突症” OR “椎间盘膨出” OR “腰椎间盘膨出” OR “腰椎椎间盘突出”OR “下腰疼” OR “腰椎间盘脱出症” OR “腰痛” OR “腰椎间盘脱出” OR “腰背痛” OR “腰间盘突出症” OR “椎间盘突出症” OR “腰椎间盘突出” OR “腰间盘突出”) AND (“系统评价” OR “系统综述” OR “元分析” OR “荟萃分析” OR “meta”) | |
| **Database 8 Chongqing VIP** | |
| (M=瑜伽) AND (M=下腰痛+腰椎间盘突出症+腰背疼痛+腰椎盘突出症+腰椎盘突出+椎间盘突出+腰腿痛+下腰痛+下背痛+腰椎间盘纤维环破裂症+腰突症+椎间盘膨出+腰椎间盘膨出+腰椎椎间盘突出+下腰疼+腰椎间盘脱出症+腰痛+腰椎间盘脱出+腰背痛+腰间盘突出症+椎间盘突出症+腰椎间盘突出+腰间盘突出) AND (M=系统评价+系统综述＋元分析＋荟萃分析＋meta) | |
| **Database 9 Sino-Med** | |
| ("瑜伽"[不加权:扩展] ) AND("腰痛"[不加权:扩展] OR "椎间盘移位"[不加权:扩展]) OR "下腰痛"[常用字段:智能] OR "腰椎间盘突出症"[常用字段:智能] OR "腰背疼痛"[常用字段:智能] OR "腰椎盘突出症"[常用字段:智能] OR "腰椎盘突出"[常用字段:智能] OR "椎间盘突出"[常用字段:智能] AND "腰腿痛"[常用字段:智能] AND "下背痛"[常用字段:智能] AND "腰痛"[常用字段:智能])AND("Meta分析"[不加权:扩展]OR "系统综述"[常用字段:智能] OR "系统评价"[常用字段:智能] OR "元分析"[常用字段:智能] OR "荟萃分析"[常用字段:智能] OR "meta"[常用字段:智能]) | |
