# Supplementary material for: Efficacy and safety of yoga for the management of chronic low back pain: an overview of systematic reviews
Source: Front Neurol. 2023 Oct 27;14:1273473. doi: 10.3389/fneur.2023.1273473 (PMC10641484; doi:10.3389/fneur.2023.1273473)
Supplement: Supplementary file 2 [file Data_Sheet_2.docx]

**Appendix 2：A protocol of efficacy and Safety of Yoga for Chronic Low Back Pain: an overview of systematic reviews**

**1.Searches**:

The literature search will be conducted by two experienced reviewers, an electronic literature search was conducted in the China National Knowledge Infrastructure (CNKI), Wan fang database (WF), China Biomedical database (CBM), Chinese Scientific Journals Database (VIP), PubMed, Cochrane Library, Web of Science (WOS), and EMBASE, the search content is a systematic review and meta-analysis of yoga therapy for chronic low back pain, and the retrieval time was from the establishment of the database to March, 2023. The search strategy will consist of both controlled vocabulary, such as the National Library of Medicine’s MeSH (Medical Subject Headings) terms, and keywords. Relevant grey literature (literature that is not published or widely available) will be identified by searching public health websites (e.g. Chinese Clinical Trial Registry).

**2.Eligibility criteria:**

- Types of Participants.

Patients who are diagnosed with chronic low back pain or chronic non-specific low back pain, according to standard diagnostic criteria.

- Types of Interventions

Yoga or combined with other therapies.

- Types of comparisons

Therapies other than yoga, such as other exercise therapy, drugs, placebo, health education, blank control, etc.

- Types of Outcomes

The primary outcome was pain, include Oswestry Disability Index scores, and Visual, Analogue Scale scores. Secondary outcome measures included disability function, quality of life, SF-36 scale, adverse effects.

- Types of Reviews

Systematic reviews with or without meta-analysis of randomized controlled trials (RCTs) were included in which yoga was used as treatment for chronic low back pain.

**3.Exclusion criteria**

- Duplicate publications;
- Non-RCTs included in MAs/SRs;
- Overviews, meetings, protocols, meta-analyses of animal experiments.
- Non-full text.

**4.Data extraction**

According to the pre-set inclusion and exclusion criteria, two evaluators will screen the preliminarily searched literature by reading the title, abstract or full text. Excel 2019 will be used to independently extract data, the results of data extraction will be cross-checked by the 2 reviewers to verify their accuracy. In case of disagreement, the third reviewer (SL) will participate in the discussion and make the final decision, and study selection will be reported using the PRISMA flow diagram.

**5. Quality appraisal and assessment of evidence**

Two trained and qualified reviewers used the Assessing the Methodological Quality of Systematic Reviews 2 (AMSTAR-2), Preferred Reporting Item for Systematic Review and Meta-analysis-2020(PRISMA-2020), and Grading of Recommendations Assessment, Development and Evaluation (GRADE) to assess the methodological, reporting, and evidence quality of the included studies, respectively. Any disagreements between the reviewers were resolved through consultation with an experienced, authoritative third reviewer. In addition, the CCA value (corrected coverage area) is used to evaluate the degree of overlap included in SRs.

**6. Data synthesis**

The results of the literature search and screening will be summarized descriptively, summarize all the results included in the SRs/MAs through a narrative description, and analyze the reliability of the conclusions based on the quality assessment results.

**Anticipated or actual start date**

July 2022
